# The complete chloroplast genome of an endangered species *Apostasia ramifera* (Orchidaceae)

**DOI:** 10.1080/23802359.2021.1872429

**Published:** 2021-02-09

**Authors:** Fang Zheng, Jian-Bing Chen, Wei-Rong Liu, Meng Wang

**Affiliations:** a Key Laboratory of National Forestry and Grassland Administration for Orchid Conservation and Utilization, The Orchid Conservation and Research Centre of Shenzhen, Shenzhen, China;; b Shenzhen Key Laboratory for Orchid Conservation and Utilization, The National Orchid Conservation Centre of China and The Orchid Conservation and Research Centre of Shenzhen, Shenzhen, China

**Keywords:** *Apostasia ramifera*, *Apostasia*, chloroplast genome, phylogenetic analysis

## Abstract

*Apostasia ramifera* S. C. Chen & K. Y. Lang 1986 is a Chinese endemic and endangered orchid. Here, we report the complete chloroplast (cp) genome sequence and the cp genome features of *A. ramifera*. The cp genome was 157,518 bp in length with a typical quadripartite structure, which was comprised of one large single-copy region (LSC, 86,353 bp) and one small single-copy region (SSC, 16,445 bp) separated by two inverted repeat regions (IRs, 27,360 bp). The cp genome encoded 133 genes, which included 87 protein-coding genes, 38 tRNAs and eight rRNAs. The average GC content of the genome is 35.8%. The phylogenetic analysis showed that *A. ramifera* was sister with *A. wallichii* and then nested in the other *Apostasia* species.

Apostasioideae is a special subfamily of Orchidaceae, which contains only two genera (*Apostasia* and *Neuwiedia*) and has several unique traits differing from other orchids, such as possessing a non-resupinate and actinomorphic flower with an undifferentiated labellum (Zhang et al. 2017). The unique features of Apostasioideae make it become important for the research of orchids evolution history. *Apostasia ramifera*, only distributed in Hainan, is one representative and Chinese endemic species of the genus *Apostasia*, which includes only eight species in the world (Govaerts et al. 2020).

Chloroplast genomes have heightened our understanding of plant on diversity and evolution, providing us more information to species identification and genetic engineering. However, the complete chloroplast genomes of only three *Apostasia* species were available in the National Center for Biotechnology Information (NCBI), including *A. wallichii*, *A. shenzhenica* and *A. odorata*. The complete chloroplast genome of *A. ramifera* was still not reported. Here, we report the first complete chloroplast genome of *A. ramifera*.

We collected the leaf samples of *A. ramifera* from the Orchid Conservation and Research Center of Shenzhen to extract genomic DNA. The specimen of *A. ramifera* was deposited in the National Orchid Conservation Center herbarium (contact person: Wenhui Rao, 359670101@qq.com) under the voucher number Z.J.Liu 6475, and DNA sample was properly stored at Key Laboratory of National Forestry and Grassland Administration for Orchid Conservation and Utilization (accession number: 6475). The modified CTAB procedure of Doyle and Doyle (1987) was employed to extract the total genomic DNA from fresh material. Genomic DNA were sequenced on Illumina Hiseq 2500 platform (San Diego, CA). Then, the obtained genome sequences were delivered to screen out and assemble with MITObim v1.8 (Hahn et al. 2013), resulting in the complete chloroplast genome of *A. ramifera* with a length of 157,518 bp. The chloroplast genome was then annotated using CPGAVAS 2 (Shi et al. 2019), then sent to Geneious Prime 2019.0.3 (https://www.geneious.com) to check and correct artificially.

The chloroplast genome sequence of *A. ramifera* (GenBank accession MT864006) was 157,518 bp in length with a typical quadripartite structure, which was comprised of one large single-copy region (LSC, 86,353 bp) and one small single-copy region (SSC, 16,445 bp) separated by two inverted repeat regions (IRs, 27,360 bp). The cp genome encoded 133 genes, which included 87 protein-coding genes, 38 tRNAs and eight rRNAs. The average GC content of the genome is 35.8%.

To confirm the phylogenetic position of *A. ramifera*, we downloaded seven chloroplast genomes from NCBI to construct a phylogenetic tree, including three *Apostasia* species, two *Neuwiedia* species and two *Vanilla* species. The two *Vanilla* species were used to root the tree. These sequences were aligned using HomBlocks (Bi et al. 2018). These aligned sequences were then sent to RAxML 8.0.0 (Stamatakis 2014) to conduct a Maximum likelihood (ML) phylogenetic tree with 1000 bootstrap replicates. We could infer from the phylogenetic tree that *A. ramifera* was most related taxa with *A. wallichii* and then nested in the other *Apostasia* species (Figure 1).

**Figure 1.**
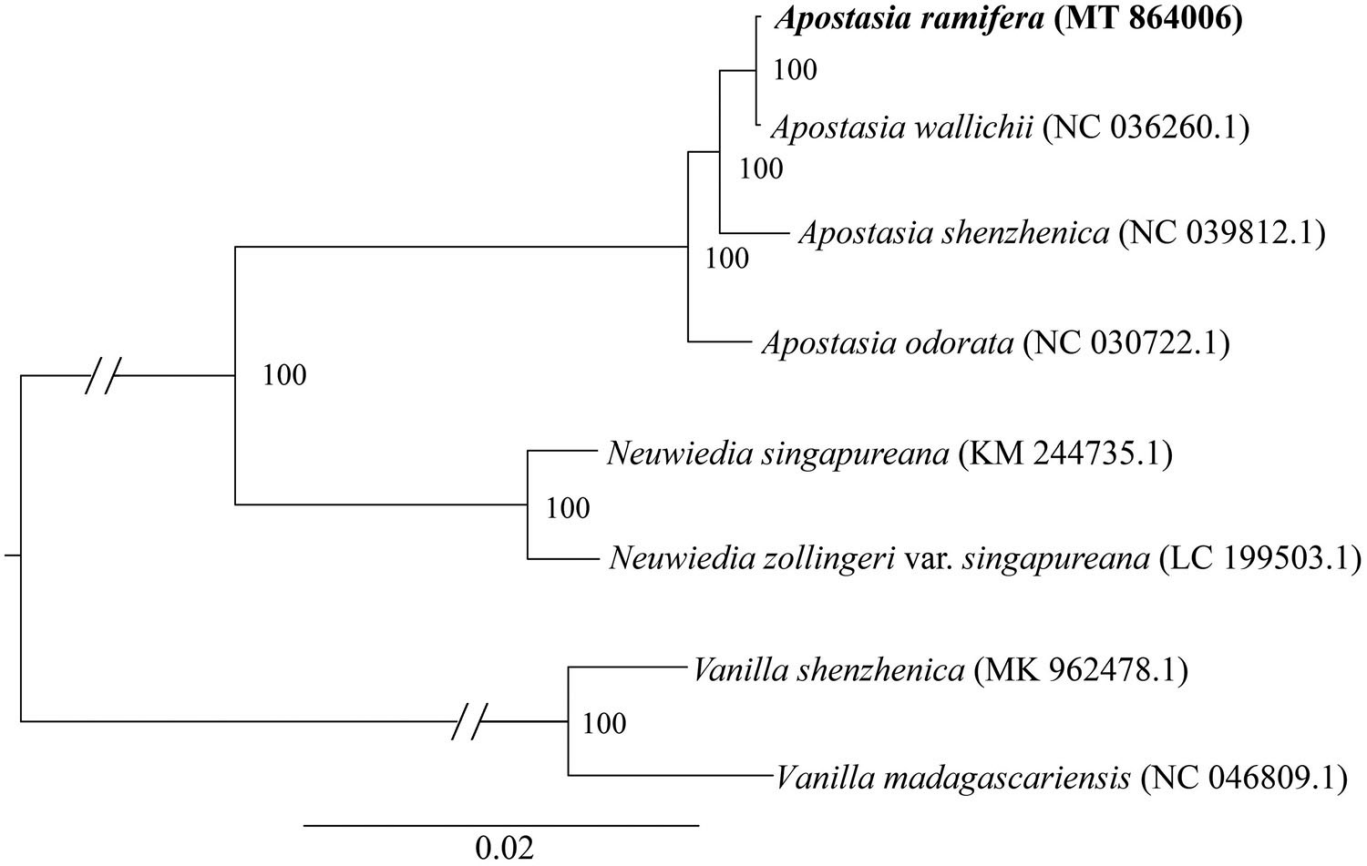
Phylogenetic position of *Apostasia ramifera* inferred by maximum likelihood (ML) of complete cp genome. The bootstrap values are shown next to the nodes.

## Data Availability

The genome sequence data that support the findings of this study are openly available in GenBank of NCBI at https://www.ncbi.nlm.nih.gov/ under the accession no. MT864006. The associated ‘BioProject,’ ‘SRA,’ and ‘Bio-Sample’ numbers are PRJNA664626, SRR12680893, and SAMN16227106 respectively.
